# Occupational Physical Activity and Regular Exercise Are Inversely Correlated with Thyroid Function in Patients with Hashimoto’s Thyroiditis

**DOI:** 10.3390/diseases12110281

**Published:** 2024-11-06

**Authors:** Marko Vuletić, Dean Kaličanin, Ana Barić Žižić, Maja Cvek, Sanda Sladić, Veselin Škrabić, Ante Punda, Vesna Boraska Perica

**Affiliations:** 1 Department of Nuclear Medicine, University Hospital of Split, Spinčićeva 1, 21000 Split, Croatia; mavuletic@gmail.com (M.V.); ana.baaric@gmail.com (A.B.Ž.); maja.cvek.st@gmail.com (M.C.); sanda_gracan@yahoo.com (S.S.); ante.punda@mefst.hr (A.P.); 2 Department of Medical Biology, School of Medicine, University of Split, Šoltanska 2, 21000 Split, Croatia; dkalican@mefst.hr; 3 Department of Paediatrics, University Hospital of Split, Spinčićeva 1, 21000 Split, Croatia; veselin.skrabic@mefst.hr

**Keywords:** autoimmunity, Hashimoto’s thyroiditis, occupational physical activity, recreational exercise, vitamin D

## Abstract

Objective: We evaluated correlations of occupational physical activity (OPA) and recreational exercise (RE), respectively, with thyroid function in patients with Hashimoto’s thyroiditis (HT). Methods: We included 438 individuals with clinically diagnosed HT. Information on OPA and RE were collected through a self-report questionnaire. We assessed correlations between clinical phenotypes (TSH, T3, T4, fT4, TgAb, TPOAb, thyroid volume, vitamin D) and physical activities (OPA and RE) in all HT patients (ALL) and in two severity-based subgroups of patients (MILD and OVERT). Results: The main novel findings are significant correlations between increase in OPA and (i) a decrease in fT4 (OVERT, r = −0.265, *p* = 0.0002 and ALL, r = −0.138, *p* = 0.006); (ii) an increase in TSH (ALL, r = 0.124, *p* = 0.014 and OVERT, r = 0.183, *p* = 0.013) and (iii) an increase in TPOAb antibodies (ALL, r = 0.101, *p* = 0.045). In contrast, we observed correlations between increase in RE and: (i) a decrease in TSH (OVERT, r = −0.238, *p* = 0.001); (ii) a decrease in TgAb antibodies (OVERT, r = −0.194, *p* = 0.01) and (iii) an increase in vitamin D levels (ALL, r = 0.146, *p* = 0.005 and OVERT, r = 0.173, *p* = 0.023). Conclusions: Our results suggest that, unlike RE, OPA correlates with decreased thyroid function and increased thyroid autoimmunity. Our study proposes that the PA health paradox also applies for the thyroid health.

## 1. Introduction

Hashimoto’s thyroiditis (HT) is a chronic autoimmune disease of the thyroid gland [1] and the most common form of autoimmune thyroid disease. The estimated prevalence of HT is between 5% and 10% in the general population [2]. It is considered as the most common cause of hypothyroidism in areas of sufficient iodine intake [1,3]. HT results from an interplay between risk genetic and environmental factors [4,5].

Most patients with HT have subclinical hypothyroidism [6,7] characterised by elevated thyroid-stimulating hormone (TSH) levels and normal levels of circulating thyroid hormones thyroxine (T4) and tri-iodothyronine (T3) [3] with a 5% annual development rate to overt hypothyroidism [6]. Clinically, HT may affect nearly all organ systems and can present itself with a variety of non-specific symptoms, most commonly fatigue, lethargy, weight gain and constipation [1,8]. Another marked feature of HT is the presence of anti-thyroidperoxidase (TPOAb) and/or anti-thyroglobulin (TgAb) antibodies [1].

Physical inactivity is a well-established risk factor for a number of medical conditions. It is considered as a global health problem [9]. Individuals with hypothyroidism are found to be less physically active than healthy adults [10], and decreased thyroid function was associated with symptoms of fatigue, muscle weakness and prolonged recovery post-exercise [11]. Diminished physical capacity is frequent in patients with hypothyroidism and it has been suggested that lower physical capacity and symptoms may persist even after introduction of supplemental levothyroxine (LT4) treatment [10,11,12].

Physical activity (PA) is a broad measure for several types of body movements, such as aerobic, muscle-strengthening activity, bone-strengthening activity and stretching, each resulting in energy expenditure [13]. PA is usually classified into categories that include occupational activities, sports, conditioning, household activities and other activities [13]. There is no single way to measure PA, but there are several frequently used methods, such as using questionnaires, diaries, accelerometers and pedometers [14]. Each of these methods has advantages and disadvantages. In our study, we used a questionnaire to asses two types of PA, occupational physical activity (OPA) and engagement in regular exercise (RE). An OPA is an activity that is work-related and performed during an 8 h work day [15]. The basic characteristic of an OPA is that it is a mandatory activity related to work tasks and productivity with reduced opportunities for workers to rest. On the other hand, RE is PA that is voluntarily performed with the aim of improving physical fitness, allowing sufficient time for rest. RE is also planned, structured and repetitive [13].

The majority of studies of PA and thyroid function investigated the immediate effects of acute high-intensity exercise on thyroid hormones in healthy individuals or trained athletes. Most of these studies found that TSH increases immediately upon intense training [16,17,18,19] and returns to a normal state with recovery. However, some studies did not observe changes in TSH levels after the intense training [20] or found that TSH levels decrease upon training [21]. Finally, one study observed that TSH may increase or decrease depending on the PA that has been performed [22]. Long-term effects of intense training and overtraining are less known; however, it has been suggested that prolonged intense training may lead to disturbance in thyroid function as training response is closely dependent on thyroid hormones [23,24,25].

It is important to distinguish the differences in effects of highly intense training on thyroid function from the effects of regularly performed less-intense exercise. Regularly performed exercise has mostly been associated with improved thyroid function in healthy individuals and individuals with subclinical hypothyroidism [26,27,28,29], although there are some inconsistencies in results [30,31].

Likewise, it is important to distinguish the differences between the effects of willingly performed RE from the effects of compulsorily performed OPA on thyroid function. There are no studies assessing this specific question.

In summary, different categories of PA have unique determinants that can have distinct effects on overall fitness and individual health [13]. In this study, we aimed to evaluate the effects of two types of physical activities (RE and OPA) on thyroid function in a large sample of clinically diagnosed patients with HT from the Croatian Biobank of HT patients (CROHT). The second aim of our study was to evaluate if the effects of RE and OPA on thyroid function differ with regard to severity of HT.

## 2. Materials and Methods

### 2.1. CROHT Biobank

This study investigates a cohort of 438 patients diagnosed with HT, predominantly female (93.38%), sourced from the CROHT biobank. The CROHT biobank sample collection and patient recruitment were performed at the Outpatient Clinic for Thyroid Disorders within the Clinical Department of Nuclear Medicine at the University Hospital of Split, spanning from 2013 to 2017.

Patient enrolment was conducted by nuclear medicine specialists following standard clinical practice and guidelines [7]. Blood samples were drawn from all participants and serum samples were preserved at −80 °C. Thyroid hormones (e.g., TSH, FT4, FT3) and thyroid-specific antibodies were measured. Comprehensive clinical assessments were conducted and thyroid ultrasound evaluations were performed to assess gland morphology and pathology. Detailed personal anamnesis was recorded and participants completed questionnaires covering various lifestyle aspects. Detailed information on the formation of the CROHT biobank, diagnostic criteria and the collection methods of various phenotypes can be found in our previously published studies [32,33,34].

### 2.2. Physical Activity

We collected information on PA through a self-report questionnaire. To assess OPA, we asked about PA during daily work, where the respondents choose one of four answers (sedentary, light, moderate and strenuous). For the purposes of OPA data analysis, we converted the four responses into scores as follows: sedentary (converted to 0), light (converted to 30), moderate (converted to 60) and strenuous (converted to 120). To assess RE, there were three questions in the questionnaire: (1) Do you play sports or exercise regularly? (2) If you exercise, how often? (3) How many hours a day do you exercise? The scoring system for RE is shown in Table 1. Participants who did not report any sports activities and exercises were scored with 0.

### 2.3. Ethics

Each participant gave explicit consent for participation in the study and for usage of their samples in future studies by signing an Agreement for Participation. The study received ethical approval from two primary bodies: The Ethics Committee of University of Split School of Medicine and the Ethics Committee of University Hospital of Split.

### 2.4. Statistical Analyses

The required sample size was calculated before conducting statistical analyses (source: https://www.stat.ubc.ca/~rollin/stats/ssize/, accessed on 22 January 2024). To achieve a study power of 80% with a 0.05 significance level (type I error), our research needed a minimum of 54 participants based on the mean and sigma of our group of patients with HT.

We used Spearman Rank correlation test to check the correlations between thyroid-related clinical phenotypes (TSH, T3, T4, fT4, TgAb, TPOAb, thyroid volume, vitamin D levels) and two types of PA: OPA and RE. We performed analyses in the whole group of 438 patients with HT (ALL), and in the two subgroups in whom patients were separated on the basis of the severity of their disease at the time of recruitment (MILD and OVERT). The MILD group included euthyroid patients and patients in subclinical hypothyroidism (TSH between 3.6 and 10 mIU/L). The OVERT group included patients in overt hypothyroidism (TSH greater than 10 mIU/L) and patients on LT4 therapy. We performed additional sensitivity analyses in two types of patients from the OVERT group separately, one in patients with TSH > 10 mIU/L without therapy and the other in patients treated with LT4 therapy. Prior to performing correlation analysis, we adjusted phenotypes for age, gender and BMI using a linear regression model. We additionally adjusted phenotypes for LT4 therapy status (yes/no) in the group of ALL HT patients and for LT4 therapy dose in the OVERT subgroup. Vitamin D levels were additionally adjusted for seasonality of blood sampling and smoking status. We used Bonferroni corrected *p*-value of less than 0.006 (corrected for eight phenotypes) for statistical significance.

We were also interested in examining which one of two types of PA is more associated with TSH status. We used a linear regression model in which TSH was selected as the dependent variable and the two PAs along with age, sex and BMI, as independent variables. In the group of ALL, therapy status was included as an additional covariate, while in the subgroup OVERT, LT4 therapy dose was included as an additional covariate.

We additionally tested if OPA and RE correlate with each other using Spearman Rank correlation test and examined differences in median scores of OPA and RE, between MILD and OVERT subgroups of patients using the Mann–Whitney test. All analyses were performed using SPSS statistical software version 20 (SPSS Inc., Chicago, IL, USA).

## 3. Results

Clinical characteristics of patients with HT are shown in Table 2. The results of correlation analyses between thyroid-specific phenotypes with the two types of PAs are shown in Table 3.

### 3.1. Results of Analysis of RE and Thyroid Function

Regarding RE (Table 3), we observed statistically significant novel correlations: (i) a decrease in TSH in OVERT (r = −0.238, *p* = 0.001); (ii) an increase in vitamin D levels in ALL (r = 0.146, *p* = 0.005) and nominally significant in OVERT (r = 0.173, *p* = 0.023) and (iii) nominally significant correlation with a decrease in TgAb antibodies in OVERT (r = −0.194, *p* = 0.01).

The results of sensitivity analysis shown in Table 4 imply the following: (i) the correlation between a decrease in TSH and RE is driven by both types of patients within OVERT and (ii) the positive correlation between vitamin D and RE is by far the most visible in patients not treated with LT4 therapy and TSH > 10 mIU/L.

### 3.2. Results of Analysis of OPA and Thyroid Function

The most interesting novel findings regarding OPA are as follows: (i) statistically significant correlations between OPA and a decrease in fT4 in OVERT (r = −0.265, *p* = 0.0002) and, to a lesser extent, in ALL (r = −0.138, *p* = 0.006); (ii) nominally significant correlations with an increase in TSH in ALL (r = 0.124, *p* = 0.014) and in OVERT (r = 0.183, *p* = 0.013); and (iii) nominally significant correlation with an increase in TPOAb antibodies in ALL (r = 0.101, *p* = 0.045).

The results of the sensitivity analysis in two types of patients within the OVERT group and OPA are shown in Table 4. These results imply the following: (i) the correlation between OPA and increased TSH is driven by the group of HT patients who are not treated with LT4 therapy and have TSH > 10 mIU/L; (ii) the negative correlation between OPA and T3, and T4, respectively, is also only noticeable in the group of patients not treated with LT4 therapy and with TSH > 10 mIU/L; (iii) however, the negative correlation between OPA and fT4 is very similar between two types of patients within the OVERT group.

### 3.3. Results of Analysis of Joint Effect of RE and OPA on TSH

**Figure 1:**
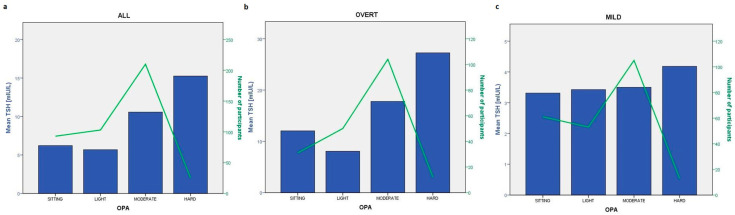
Graphical representation of the mean TSH levels across four OPA categories in three groups of patients with HT: ALL (**a**), OVERT (**b**) and MILD (**c**). Bars represent the mean TSH levels per different type of OPA and the green line represents the number of participants.

**Figure 2:**
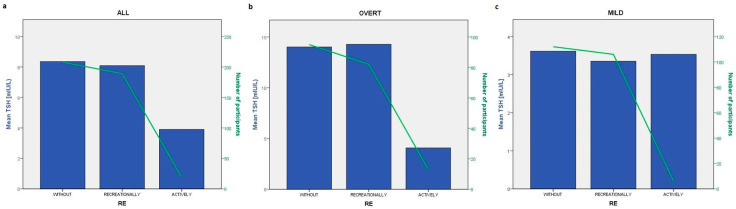
Graphical representation of the mean TSH levels across three RE categories (without, recreationally, actively) that answer the question from the questionnaire: “Do you play sports or exercise regularly?” in ALL (**a**), OVERT (**b**) and MILD (**c**). Bars represent the mean TSH levels per different type of RE and green line represents the number of participants.

A graphical representation of the mean TSH values shown in bars with respect to the OPA and RE categories is presented in Figure 1 and Figure 2, respectively. For graphical representation, RE categories were derived on the basis of the question “Do you play sports or exercise regularly” with three possible answers: without, recreationally and actively.

To analyse possible predictors of TSH, we performed linear regression analysis that showed that OPA was significantly associated with TSH levels in ALL (β = 0.086, *p* = 0.006) and OVERT (β = 0.178, *p* = 0.004), whereas RE was not significantly associated in ALL but was nominally associated in OVERT.

There was no correlation between RE and OPA in any investigated group (ALL, r = 0.052, *p* = 0.298; MILD, r = 0.039, *p* = 0.568 and OVERT, r = 0.073, *p* = 0.327), indicating that PA during work hours does not correlate with engagement in exercise.

However, we detected a significant difference in median OPA score between OVERT and MILD groups of patients with HT (*p* = 0.036) with higher score in OVERT patients. There was no significant difference in median RE score between two subgroups of patients with HT (*p* = 0.828).

## 4. Discussion

A beneficial role of exercise on thyroid function has been suggested; however, the role of work-related PA on thyroid health has not been thoroughly evaluated, especially in patients with HT. We therefore aimed to analyse and compare the impacts of these two types of physical activities on thyroid function in patients with chronic autoimmune thyroiditis. Our results demonstrate opposing effects of these two types of activities on thyroid function. Namely, we find that OPA is negatively correlated with thyroid function, while RE is positively correlated with thyroid function in patients with HT (Table 3). Additionally, to account for the heterogeneity of the study population, we analysed data in two subgroups of HT patients according to disease severity. Our results indicate that both positive and negative effects of two types of PAs are more pronounced in the subgroup of HT patients with more severe disease (OVERT).

### 4.1. RE and Thyroid Function

We observe that RE is significantly correlated with a decrease in TSH levels in OVERT patients with HT (r = −0.238, *p* = 0.001) (Table 3). As noted in the introduction, many studies have analysed the immediate effects of high-intensity exercise on thyroid-related parameters in healthy individuals or athletes. The impact of regularly performed RE on thyroid health has also been evaluated, and several studies of patients in subclinical hypothyroidism and the general population have, in agreement with our study, found a beneficial effect of RE on thyroid function. A cross-sectional study on 159 individuals with subclinical hypothyroidism from the Epidemiological Survey of Thyroid Diseases in Fujian Province found that moderate and high-intensity exercise are associated with lower TSH and better thyroid secretion [26]. Studies on the general population also found similar results. For example, a study of 5385 participants from the *She*, a population-based study of an ethnic minority in China, observed that individuals who performed physical activities for at least 20–30 min twice or more per week had significantly decreased incidence of hypothyroidism [27]. A recent study of the influence of PA on thyroid hormones in >4000 individuals from the U.S. National Health and Nutrition Examination Survey (NHANES) reported that individuals who conducted moderate and vigorous daily PA, measured by two types of questionnaires (PAD200 and binary PAD200), had lower TSH and T4 levels [28]. Moreover, the NHANES study found that physically active individuals produce less TSH at low T4 levels, suggesting that PA influences the hypothalamic–pituitary axis through effects of thyroid hormone signalling [28]. On the other hand, Dueñas et al. did not observe an association between TSH and PA in 2470 participants from the cross-sectional study, 1907 participants from the longitudinal population-based study and 219 individuals with hypothyroidism, using the LASA Physical Activity Questionnaire (LAPAQ) [31]. Hypothyroidism was defined as TSH levels greater than 4.0 mIU/L; however, no detailed clinical information was given to clarify if individuals were in subclinical or in overt hypothyroidism [31]. It is important to note that the average age of individuals included in the study (57.3 years) is higher than the average age of individuals from our study (39.3 years). Age generally affects the rate of PA, meaning that older participants usually engage in less intense PA that may consequently reduce the association with thyroid function [35,36,37].

Even though the results of the studies are not completely conclusive, the majority still indicate that regular RE may be protective against hypothyroidism in the general population and beneficial to thyroid function in patients with hypothyroidism, especially those in the OVERT stage.

This is a very useful finding, as it demonstrates that the correlation between RE and thyroid function is more pronounced in individuals with higher grades of thyroid malfunction. It is consistently shown that patients from the OVERT group have many features that differ from those in patients from the MILD group, indicating that these are two distinct entities of HT patients with regards to their symptomatology, pathophysiology and response to environmental triggers [34,38]. Our results suggest that patients with HT, especially those who are burdened with longer and more severe disease may benefit from introduction of regular exercise in addition to hormone therapy.

We also observed a nominally significant correlation between RE and decreased TgAb levels in OVERT (r = −0.194, *p* = 0.010). PA has already been shown to have immunomodulatory effects on the immune system in several autoimmune diseases [39]. Studies have shown that physically active individuals have fewer activated CD4 and CD8 T cells compared to inactive ones [40], and that aerobic exercise specifically inhibits Th1 cell activity [39]. Exercise promotes the increase in regulatory T cells [39], which play a significant role in homeostasis of the immune system and prevention of autoimmune response, and which were found to be reduced and less functional in patients with HT [4,41]. Furthermore, exercise helps prevent the accumulation of adipose tissue, which is thought to be an important source of chronic low-grade inflammation [42,43]. Finally, it has been reported that exercise can reduce immunoglobulin secretion, and β-endorphin released after exercise was shown to decrease antibody secretion [39].

Finally, we observe another statistically significant positive correlation of RE with vitamin D levels in ALL HT patients (r = 0.146, *p* = 0.005). Our results suggest that patients who regularly practice RE tend to have higher vitamin D levels. We observe this effect in the whole group of HT patients (ALL), and in OVERT subgroup. Sensitivity analysis showed that within the OVERT group, patients who are not treated with LT4 therapy and have TSH > 10 mIU/L have the greatest positive correlation between RE and vitamin D levels (Table 4).

We recently reported that our HT patients have high proportions of vitamin D deficiency and that patients from the OVERT subgroup tend to have lower vitamin D median values than patients from the MILD subgroup [34]. Our finding of a positive correlation between RE and vitamin D is important because it suggests that a simple change in lifestyle, such as conducting regular exercise, may alleviate the problem of vitamin D deficiency in patients with HT, especially those not treated with therapy and with TSH greater than 10 mIU/L. It is worth noting that our study population resides in the region with a Mediterranean climate and plenty of annual sunny days. The limitation of our study is that we have no information on the outdoors activities of our study participants. Therefore, we cannot rule out that our finding of a positive correlation between RE and higher vitamin D levels is partially due to the fact that recreational activities take place outdoors.

### 4.2. OPA and Thyroid Function

Interestingly, the majority of studies were focused on analysing the effects of leisure-time exercise on thyroid function. However, these studies did not investigate associations of other domains of PA with thyroid function, mainly the effects of PA during work hours. We were interested in evaluating this question. A similar question was assessed by the above-mentioned NHANES study. The question PAQ180 asked subjects to rate their overall activity level from 1 to 4, with each successive number being a higher activity level, similar to our study. Interestingly, in contrast to results observed when analysing questions that assessed vigorous PA (PAD200 and binary PAD200), the overall PA measured by PAQ180 was not beneficially associated with TSH or T4 [28], indicating that higher overall daily activity does not have a positive effect on thyroid function. Although the authors did not discuss these results, we can align them with our results, as we also do not observe positive impacts of OPA on thyroid function. On the contrary, we observe a negative correlation between OPA and thyroid function, as indicated by several of our results: statistically significant correlation with lower fT4 (ALL: r = −0.138, *p* = 0.006; OVERT: r = −0.265, *p* = 0.0002), nominally significant correlation with higher TSH (ALL: r = 0.124, *p* = 0.014; OVERT: r = 0.183, *p* = 0.013) and nominally significant correlation with higher TPOAb (ALL: r = 0.101 *p* = 0.045). Our sensitivity analysis indicated that the correlation between OPA and increased TSH is mainly driven by the subgroup of patients that are not treated with LT4 therapy and have TSH > 10 mIU/L. Moreover, in this group we also observe a negative correlation between OPA and T3 and T4 (Table 4).

Several other studies observed that hypothyroidism is more prevalent in individuals who work longer hours (53–83 h per week) [44] or in shift workers in comparison to daytime workers, indicating that other factors, such as long work hours, sleep disturbance and stress related to work may act as risk factors for development of autoimmune hypothyroidism [45,46]. Indeed, stress alters the hypothalamic–pituitary–thyroid (HPT) axis and has been proposed as an environmental factor for thyroid autoimmunity [47,48].

In line with our study, opposing effects of OPA and RE are very well documented with cardiovascular disease and termed the physical activity health paradox [49].

### 4.3. Joint Effect of RE and OPA on TSH

Finally, when analysing associations of both types of physical activities with TSH, our results indicated that only OPA is significantly associated with TSH. We also detected significantly higher OPA score in the OVERT subgroup of HT patients in comparison to MILD, whereas there was no significant difference in median RE score between the two subgroups. These results imply that PA during work hours has a dominant effect on thyroid function and that patients with higher OPA have more severe disease. An equivalent conclusion was drawn with regard to several other diseases (all-cause mortality, cardiovascular disease, musculoskeletal pain, diabetes, depression) indicating that the beneficial effects of RE are much less protective in individuals with moderate and high OPA [50]. Finally, a study examining cardiovascular parameters observed that OPA and leisure time PA are inversely associated with autonomic regulation during sleep and that beneficial effects of leisure time PA were diminished with higher OPA levels [51].

### 4.4. Limitations and Advantages

This is a cross-sectional study and, by design, it is limited to infer causality between the effects of two types of physical activities and thyroid function. Another limitation is that we used a self-reported questionnaire that could be prone to recall bias. Our questionnaire also did not assess information on the intensity of the PAs, which could influence the observed effects. However, the usage of a questionnaire is the most cost-effective method for collection of data on PA in cohort studies, like ours. A recent systematic review found a weak-to-moderate correlation between the use of accelerometry and PA questionnaires. The same study implied that questionnaires have advantages such as they make it easier to assess PA over longer periods of time and with them one can assess all types of physical activities [14]. One thing that is important to stress is that beside these two types of activities, there are other physical activities in between, like prolonged hikes, gardening activities and household activities, that can all affect the health of an individual and should be taken into consideration in future studies [52].

The advantages of our study include the usage of a large cohort of clinically diagnosed patients with a wide range of thyroid-related clinically important phenotypes. Another advantage is that we were able to perform sensitivity analyses in two subgroups of HT patients stratified by severity of disease using stringent diagnostic criteria. To our knowledge, this is the first study that analysed correlations between PA during work hours and thyroid function in patients with HT.

## 5. Conclusions

Our study suggests that regularly performed exercise, RE, is correlated with improvements in thyroid function of patients with HT, as evidenced by the negative correlations with TSH and TgAb and the positive correlation with vitamin D. In contrast, work-related physical activity, OPA is correlated with decreased thyroid function, as evidenced by the positive correlations with TSH and TPOAb and the negative correlation with fT4. Our study supports the already proposed physical activity health paradox and extends it to the field of thyroid function. We also observe that higher OPA activity has a dominant, negative effect on TSH in comparison with RE.

An additional message of our study is the importance of stratifying patients according to disease severity, because patients at the beginning of the disease (MILD) and those in a more severe condition (OVERT) differ in their underlying physiology and response to external stimuli. Our results indicate that HT patients with high TSH (greater than 10 mIU/L and without therapy) are the most vulnerable to the effects of high OPA.

From a clinical perspective, there is a need for development of an individual patient-management programme, in which the benefits of a reduced workload, as well as the implementation of a regular exercise program, would be considered. Further studies are warranted to determine mechanisms by which PA affects thyroid work.

## Figures and Tables

**Table 1:** Calculation of the score for recreational exercise (RE).

	Less than an Hour	Between 1 and 2 h	Over 2 h
Daily	30	45	60
2–3 times a week	10	15	20
Once a week	4	6	8
Occasionally	1	1.5	2

**Table 2:** Clinical characteristics of HT patients (ALL) and patients with HT divided in two disease-severity groups (OVERT and MILD).

Parameter	ALL	OVERT	MILD
	(N = 438)	(N = 203)	(N = 235)
	Median (Q1–Q3)	Median (Q1–Q3)	Median (Q1–Q3)
Female, N (%)	409 (93.38)	185 (91.13)	224 (95.32)
Age, years	38.45 (28.44–48.95)	41.19 (31.46–50.94)	35.72 (26.27–46.95)
BMI, kg/m^2^	23.58 (20.83–26.89)	23.97 (21.12–26.92)	23.23 (20.76–26.65)
TSH, mIU/L	3.27 (1.74–5.55)	3.34 (1.66–12.00)	3.23 (1.82–4.71)
T3, nmol/L	1.60 (1.31–1.80)	1.60 (1.30–1.80)	1.70 (1.40–1.85)
T4, nmol/L	105.00 (89.15–118.00)	103.00 (85.80–121.00)	106.00 (91.60–116.80)
fT4, pmol/L	12.10 (10.20–13.20)	12.10 (9.90–13.70)	12.10 (10.90–13.10)
TgAb, IU/mL	134.50 (36.55–422.30)	175.60 (49.30–576.05)	123.00 (26.30–327.00)
TPOAb, IU/mL	202.00 (27.03–628.75)	244.20 (63.15–881.00)	156.00 (16.95–527.55)
Thyroid volume, cm^3^	10.05 (7.34–14.25)	9.92 (6.97–15.09)	10.45 (7.72–13.25)
Vitamin D, ng/mL	19.50 (14.40–25.10)	18.45 (13.90–23.47)	20.90 (15.02–25.97)

ALL—all HT patients; MILD—HT patients that were euthyroid or in subclinical hypothyroidism, OVERT—HT patients that were in overt hypothyroidism or treated with levothyroxine (LT4) therapy; Q1—first quartile, Q3—third quartile.

**Table 3:** Correlation analysis between thyroid-related phenotypes and two types of physical activities (OPA and RE).

	Occupational Physical Activity (OPA)			Recreational Exercise (RE)		
Phenotype	ALL (N = 438)	OVERT (N = 203)	MILD (N = 235)	ALL (N = 438)	OVERT (N = 203)	MILD (N = 235)
	r (p)	r (p)	r (p)	r (p)	r (p)	r (p)
TSH	**0.124 (0.014)**	**0.183 (0.013)**	0.033 (0.629)	−0.007 (0.896)	**−0.238 (0.001)**	−0.018 (0.790)
T3	−0.079 (0.115)	−0.089 (0.228)	−0.052 (0.450)	−0.021 (0.684)	0.066 (0.386)	−0.064 (0.361)
T4	−0.028 (0.575)	−0.086 (0.244)	0.039 (0.571)	−0.051 (0.325)	0.077 (0.310)	−0.093 (0.182)
fT4	**−0.138 (0.006)**	**−0.265 (0.0002)**	−0.043 (0.543)	−0.030 (0.560)	0.075 (0.324)	0.013 (0.849)
TgAb	0.029 (0.571)	0.016 (0.826)	−0.008 (0.911)	0.002 (0.975)	**−0.194 (0.010)**	0.108 (0.120)
TPOAb	**0.101 (0.045)**	0.092 (0.214)	0.048 (0.487)	0.057 (0.271)	−0.055 (0.465)	0.077 (0.267)
Thyroid volume	0.078 (0.132)	0.065 (0.396)	0.074 (0.293)	0.049 (0.359)	−0.047 (0.549)	0.060 (0.398)
Vitamin D	0.089 (0.081)	0.064 (0.395)	0.099 (0.154)	**0.146 (0.005)**	0.173 (0.023)	0.124 (0.081)

r—Spearman’s correlation coefficient; adjusted for age, gender, BMI and LT4 therapy status (yes/no); OPA—occupational physical activity; RE—recreational exercise; Vitamin D—additional adjustments for seasonality of blood sampling and smoking status; statistically significant and nominally significant results are shown in bold.

**Table 4:** Correlation analysis between thyroid-related phenotypes and two types of physical activities (OPA and RE) in two types of patients within the OVERT group.

	OPA		RE	
Phenotype	TSH > 10 (N = 46) NT	OT (N = 157)	TSH > 10 (N = 46) NT	OT (N = 157)
	r (p)	r (p)	r (p)	r (p)
TSH	0.387 (0.014)	−0.012 (0.887)	−0.153 (0.373)	−0.084 (0.297)
T3	−0.363 (0.021)	0.026 (0.753)	−0.048 (0.780)	0.084 (0.322)
T4	−0.377 (0.017)	0.041 (0.622)	−0.035 (0.837)	0.050 (0.559)
fT4	−0.274 (0.091)	−0.215 (0.010)	0.108 (0.537)	0.041 (0.632)
TgAb	0.020 (0.901)	−0.126 (0.133)	−0.227 (0.183)	0.035 (0.679)
TPOAb	−0.104 (0.523)	0.106 (0.207)	−0.221 (0.196)	0.005 (0.954)
Thyroid volume	0.152 (0.364)	0.027 (0.759)	−0.011 (0.950)	−0.063 (0.476)
Vitamin D	−0.030 (0.856)	0.090 (0.291)	0.467 (0.004)	0.071 (0.409)

r—Spearman’s correlation coefficient; adjusted for age, gender, BMI and LT4 therapy dose; Vitamin D—additional adjustments for seasonality of blood sampling and smoking status. NT—patients not undergoing LT4 therapy; OT—patients on LT4 therapy.

## Data Availability

The data supporting the conclusions of this article will be made available by the corresponding author upon reasonable request.
